# PTEN and Gynecological Cancers

**DOI:** 10.3390/cancers11101458

**Published:** 2019-09-28

**Authors:** Camilla Nero, Francesca Ciccarone, Antonella Pietragalla, Giovanni Scambia

**Affiliations:** Gynecologic Oncology, Dipartimento per le Scienze della Salute della Donna, Fondazione Policlinico Universitario A. Gemelli IRCCS, del Bambino e di Sanità Pubblica, 00168 Roma, Italy; fr.ciccarone@gmail.com (F.C.); antonella.pietragalla@policlinicogemelli.it (A.P.); giovanni.scambia@policlinicogemelli.it (G.S.)

**Keywords:** PTEN, ovarian cancer, endometrial cancer, cervical cancer, vulvar cancer, uterine cancer

## Abstract

PTEN is a tumour suppressor gene, and its loss of function is frequently observed in both heritable and sporadic cancers. It is involved in a great variety of biological processes, including maintenance of genomic stability, cell survival, migration, proliferation and metabolism. A better understanding of PTEN activity and regulation has therefore emerged as a subject of primary interest in cancer research. Gynaecological cancers are variously interested by PTEN deregulation and many perspective in terms of additional prognostic information and new therapeutic approaches can be explored. Here, we present the most significant findings on PTEN in gynaecological cancers (ovarian, endometrial, cervical, vulvar and uterine cancer) focusing on PTEN alterations incidence, biological role and clinical implications.

## 1. Background

Molecular characterization of cancer provides critical information for clinical oncology. A quick paradigm shift is therefore occurring in cancer prevention, treatment and follow up. Gynaecological malignancies make no exception. Overall, the capability of sustaining proliferative signalling is considered the most fundamental trait of cancer cells. In particular, the phosphatidylinositol 3-kinase (PI3K) pathway operates at multiple nodes within the proliferative signalling circuitry. Phosphatase and tensin homolog deleted on chromosome 10q23.3 (PTEN) is mainly involved as a negative regulator in the PI3K/AKT/mTOR pathway through both enzymatic and non-enzymatic activities and PI3K-dependent and -independent mechanisms and its deregulation occurs in a large proportion of human heritable and sporadic cancers [1]. 

Many in vitro studies have revealed that this tumour suppressor gene is involved in an intricated network of signalling effectors regulating several crucial cell functions such as proliferation, survival, genomic stability and cell motility [2,3,4]. 

In particular, PTEN contains two key domains for its tumour suppression function: the phosphatase domain and the C2 domain. As a tumour suppressor it acts through its 3’-phosphatase action dephos-phorylating phosphatidylinositol (3,4,5)-trisphosphate (PIP_3_) resulting in an inhibition of AKT and hence the rest of the signalling cascade. More in detail, the PTEN protein has both lipid phosphatase activity, involved in the arrest of cell-cycle progression at G1/S, and protein phosphatase activity, involved in the inhibition of focal adhesion formations, cell spreading, and migration, as well as the inhibition of growth factor-stimulated MAPK signalling. The combination of the losses of both phosphatase activities may result in aberrant cell growth and an escape from apoptosis, as well as abnormal cell spreading and migration [5,6].

PTEN acts also as a scaffold protein in both the nucleus and cytoplasm through a phosphatase-independent function in the nucleus serving as a chromosomal stability controller [4]. 

Across 4 of the more frequent cancers in women (breast, ovarian, endometrial and cervical cancers), PTEN has been shown to be one of the most frequently mutated genes (13%) [7].

In this review, we illustrate the incidence, the role in tumorigenesis and the clinical implications of PTEN deregulation in ovarian cancer (OC), endometrial cancer (EC), cervical cancer (CC), vulvar cancer (VC) and uterine cancer (UC), including mainly uterine carcinosarcoma/uterine malignant mixed mullerian tumour and leiomyosarcoma.

## 2. Overview 

Considering the incidence of PTEN alterations (including all somatic chromosomal abnormalities and gene mutations) across human cancers, the highest percentages are found in endometrium, central nervous system (glioblastoma), skin, and prostate cancers according to the Catalogue of Somatic Mutations in Cancer (COSMIC) and The Cancer Genome Atlas Network (TGCA) datasets [8,9]. 

COSMIC is currently the most comprehensive global resource for information on somatic mutations in human cancer; it contains reanalysed data from the scientific literature, the Cancer Genome Project’s systematic screens, and data from the International Cancer Genome Consortium and TCGA.

TGCA molecularly characterized over 20,000 primary cancers and matched normal samples spanning 33 cancer types. 

Although main clinical information is provided in both datasets, data are still quite heterogeneous.

**Figure 1:**
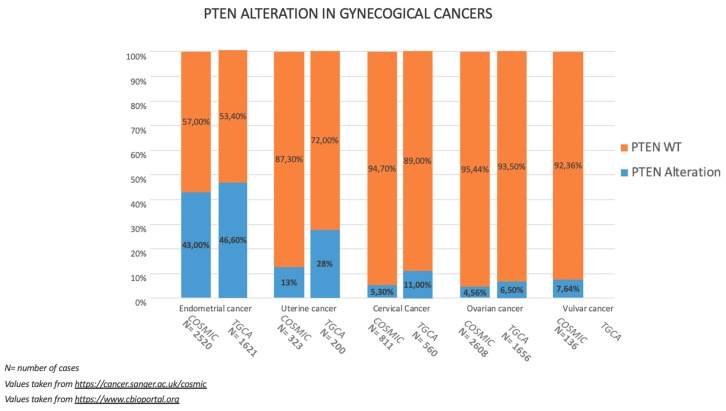
The incidence and type of PTEN alterations according to COSMIC and TGCA datasets in gynecological malignancies.

The incidence of somatic PTEN alteration in gynaecological cancers is illustrated in Figure 1 according to COSMIC and TGCA datasets, respectively. 

Median PTEN alteration rate is 45% in EC (43% from COSMIC and 46.6% from TGCA), 5,5% in OC (4.56% from COSMIC and 6.5% from TGCA), 8% in CC (5,3% from COSMIC and 11% from TGCA), 7.64% in VC (data available only from COSMIC datasets), 20.5% in UC (13% from COSMIC and 28% from TGCA) [8,9].

Data on EC were expected, given the fact that >80% of EC cases have at least one somatic alteration that affects signalling pathways, and the PI3K/AKT signalling pathway is one of the most frequently altered. 

PTEN alterations are much less present in the other gynaecological malignancies (OC, CC; VC and UC) but when they occur, seem to play a not negligible role in the development and progression of cancer.

## 3. Ovarian Cancer

### 3.1. Incidence of PTEN Alteration

**Figure 2:**
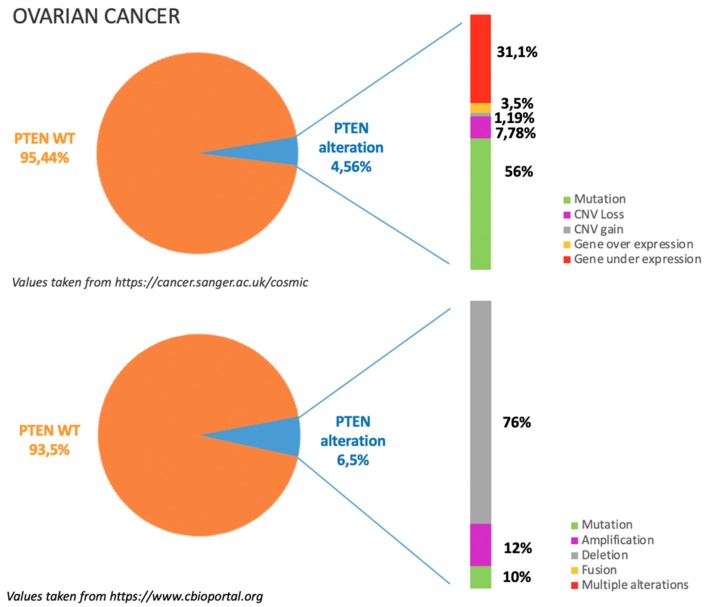
The incidence and type of PTEN alterations according to COSMIC and TGCA datasets in ovarian cancer.

As previously mentioned, PTEN alterations in OC are around 6%, mostly due to gene deletion as illustrated in Figure 2. According to COSMIC dataset, see https://cancer.sanger.ac.uk/cosmic in Figure 2, gene mutations occur in almost half of the cases in which PTEN is altered; among gene mutations missense substitutions are reported to be the most frequent (50%) followed by frameshift deletions (20.21%) and nonsense substitutions (18.09%). Around 30% of PTEN alterations are related to gene under expression. This data is consistent with TGCA dataset, see https://www.cbioportal.org in Figure 2, which reports that among PTEN alterations more than 70% are due to gene deletion [8,9,10].

Incidence of PTEN alterations is reported in Table 1 according to histological subtypes.

Kurman and Shih’s “dualistic” model of epithelial OC pathogenesis divides OC into two main categories, type I (low-grade, relatively indolent and genetically stable tumours that typically arise from recognizable precursor lesions, such as endometriosis (or so-called borderline) and type II (high-grade serous carcinomas, virtually all of which harbour mutant TP53 alleles) [11]. Type I OCs account for 30% of all epithelial OC, frequently harbour somatic mutations such as PTEN and include most endometrioid, clear cell, and mucinous carcinomas and low-grade serous carcinomas. Somatic mutations in PTEN are more common in endometroid OC. 

Japanese data on clear cell OC are consistent with TGCA and COSMIC data (5% of PTEN mutation rate analysing 39 patients) [12]. A recent publication on a phase II trial of oral ENMD-2076 in clear cell OC, shows a 44% rate (16 out of 36 patients) of PTEN loss analysed by immunohistochemistry [13]. 

An analysis focused on 22 Japanese endometriosis-related ovarian carcinomas (13 endometrioid, and nine clear cell) revealed a 68.4% PTEN mutation rate [14]. Larger numbers of this rare condition need to be analysed to confirm PTEN role in the progression of endometriosis-related ovarian carcinogenesis but available evidences seem to suggest so [15,16,17].

In contrast, type II OCs account for 70% of all epithelial OC and include mainly high-grade serous carcinomas, virtually all of which harbour mutant TP53 alleles.

Despite COSMIC and TGCA datasets show superimposable PTEN alteration rates, other evidences suggest that in high-grade serous OC these values may have been underestimated. 

It has been shown in fact that stromal DNA strongly biases estimates of PTEN expression. Over 30% of high grade serous OC tumours in fact, present with loss of PTEN when the stromal DNA contribution is excluded [18]. Moreover, immunohistochemistry studies of PTEN protein levels demonstrated total loss of PTEN in 15% (including stroma) of 52 out of the 316 TCGA tumours [19]. 

### 3.2. PTEN Pathways in OC Tumorigenesis

It is well known that Type I tumours are genetically stable and are characterized by mutations in a number of different genes including KRAS, BRAF, PTEN, and beta-catenin [11,20,21]. 

In particular, KRAS and BRAF are mutually exclusive mutations, occur in about two thirds of type I OC and result in constitutive activation of the mitogen activated protein kinase signal transduction pathway which contributes very early to neoplastic transformation [22]. 

PTEN mutations can co-exist and lead to PI3K/Akt/mTOR pathway aberrantly activation; the combination of PTEN mutations with KRAS ones in the ovary has been shown to induce invasive and widely metastatic endometrioid OC [23].

Several published pre-clinical models suggest that the loss of PTEN in the fallopian tube is one of the multiple genetic modification that induce tumorigenesis [24,25,26]. It has been then demonstrated that the conditional homozygous knockout of PTEN mediated by PAX8-cre recombinase is sufficient to drive endometrioid and serous borderline OC in mice models [27]. 

High grade serous OC has believed to have a different pathogenesis. The current model of high grade serous OC development in fact, supported by genomic, transcriptomic, and proteomic studies, indicates that it originates in the fallopian tube [28]. A key step in this process are mutations in the p53 gene leading to protein stabilization in the fallopian tube. Eventually, other tumour suppressors are lost or oncogenes amplified (e.g., PTEN and KRAS, respectively), resulting in a serous tubal intraepithelial carcinoma which subsequently metastasizes to the ovary and peritoneum [29].

The loss of PTEN has been found in four out of 12 (33%) samples of serous intratubal carcinoma [30]. According to the authors, this data suggested that alterations in the expression of PTEN, as well as PAX2, could be involved in the early stages of serous carcinogenesis, similar to endometrioid carcinomas of the uterus [31]. It is, however, unknown whether these alterations occur before malignancy, as seen in the endometrial model, or are coincident with the emergence of malignancy.

Moreover, it was recently shown that the loss of PTEN allows the fallopian tube epithelia to form multicellular tumour spheroids, which survive better under ultra-low adhesion conditions, attach to the extracellular matrix exposed during ovulation, and colonize the ovary maybe contributing to seeding of the ovary in high grade serous OC patients [32].

In fact, PTEN deletion has been associated with the acceleration of cell proliferation and cellular transformation in vitro and in vivo and the stimulation of MUC1 expression that is known to be involved in tumour cell migration and metastasis [33]. 

### 3.3. Clinical Implications

Although pathogenic variants in PTEN do not appear to confer a significantly increased risk for OC, in patients affected by PTEN hamartoma tumour syndromes, it can be discussed the potential risks and benefits of risk reducing bilateral salpingo-oopherectomy in case of who a family history of OC [34]. 

The prognostic and predictive value for PTEN in OC is still uncertain [35,36]. Its inactivation in high grade serous OC has been shown to contribute to acquired chemotherapy resistance [37].

PTEN mutation is an inclusion criterion in nine clinical trials for OC (NCT01458067, NCT01226316 and NCT02660034 phase 1; NCT02583542 and NCT02797964 phase 1–2, NCT01283035, NCT03742895 and NCT03759600 phase 2; NCT02392676 phase 3), an inclusion criteria in a subprotocol of a clinical trial (NCT02465060, phase 2) and part of the rationale in another one (NCT01989546 phase 1–2) [38]. 

The reported prevalence of deleterious somatic PIK3CA and/or PTEN variants in germline mutation-negative OC individuals (13%), together with encouraging data from Phase 1 trials on gynaecological patients receiving PI3K/AKT/mTOR inhibitors, suggest that this pathway will be more investigated in the future [39,40].

## 4. Endometrial Cancer

### 4.1. Incidence of PTEN Alteration

**Figure 3:**
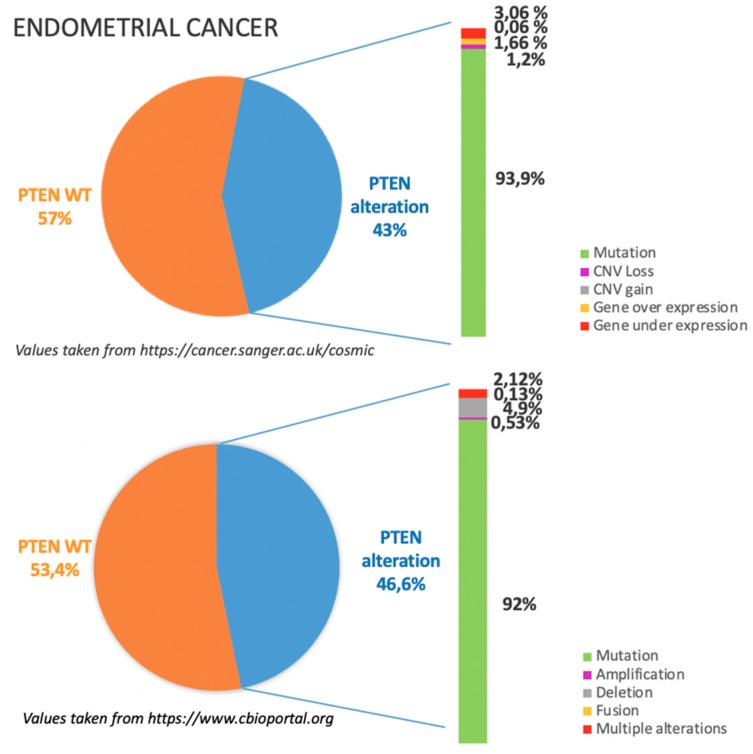
The incidence and type of PTEN alterations according to COSMIC and TGCA datasets in endometrial cancer.

As previously mentioned, PTEN alterations in EC are around 45%, mostly due to missense and nonsense substitutions as illustrated in Figure 3.

According to COSMIC dataset, see https://cancer.sanger.ac.uk/cosmic in Figure 3, gene mutations occur in the vast majority of the cases in which PTEN is altered; among gene mutations missense substitutions are reported to be the most frequent (43.84%) followed by frameshift deletions (28.47%) and nonsense substitutions (22.95%). 

TGCA dataset, see https://www.cbioportal.org in Figure 3, show superimposable results; missense substitutions are reported to be the most frequent (30.89%) followed by frameshift deletions (15.17%) [8,9].

Incidence of PTEN alterations is reported in Table 2 according to histological subtypes.

EC has been historically classified into two main clinicopathological and molecular types: type I (mainly endometrioid adenocarcinoma, accounting for 80%–90% of all EC) and type II (non-endometrioid subtypes such as serous, clear-cell and undifferentiated carcinomas, as well as carcinosarcoma/malignant-mixed Müllerian tumour, accounting for 10%–20% of all EC) [41]. Type I tumours are preferentially associated with genetic alterations in PTEN, KRAS, CTNNB1 and PIK3CA and MLH1 promoter hypermethylation, whereas type II, especially serous histotypes, prototypically harbour TP53 mutations [42].

Among the four molecular categories of EC identified by the Cancer Genome Atlas Research Network (‘ultramutated’, ‘hyper-mutated’ and ‘copy number low’, which are predominantly endometrioid, and ‘copy number high’, most of which are serous), PTEN mutations were found in 94%, 88%, 77% and 15%, respectively [42].

### 4.2. PTEN Pathways in EC Tumorigenesis

Several prior reports suggest that PTEN mutations occur early in the neoplastic process of Type I EC tumours [43,44]. 

In animal models PTEN knockout mice develop endometrial cancer precursors and cancer and women with Cowden’s disease, who carry germline PTEN mutations, are at elevated risk of EC [45,46]. 

Loss of PTEN protein expression in normal endometrial gland has been identified by immunohistochemistry in healthy premenopausal women (43% of samples) [47]. 

Under the influence of growth promoting stimuli such as excessive exposure to estrogens relative to progesterone, endometrial proliferation is favoured over differentiation and apoptosis, and normal glands with PTEN loss may undergo clonal expansion and develop cancer precursor and cancer.

As expected, in a recent metanalysis, PTEN loss in endometrial hyperplasia has been significantly associated with increased risk of EC (OR of 3.32; 95% CI 1.59–6.97, *p* = 0.001) [48]. 

### 4.3. Clinical Implications

In the 2016 ESMO-ESGO-ESTRO Consensus Conference on Endometrial Cancer, the immunohistochemical assessment of PTEN has been recommended to recognize premalignant endometrial hyperplasia, which often shows a loss of expression of the protein [49]. However, recent data suggest that this diagnostic method could be not accurate enough [48].

Patients affected by Cowden syndrome who carry a germline pathogenic variants in the PTEN gene, have elevated risks for breast, endometrial, and thyroid cancer [50]. Most cancers are diagnosed premenopausally between the ages of 38 and 46 years [51]. For these patients survellaince programmes are offered [52].

PTEN is an inclusion criterion in 5 clinical trials for EC (NCT01226316 and NCT01458067 phase 1, NCT03675893 and NCT02549989 phase 2, NCT02189174 phase 2, closed) and an inclusion criteria in subprotocols in two clinical trials (NCT02583542 phase 1/2, NCT02465060 phase 2). 

Moreover, in five other clinical trials (NCT03016338, NCT02990728, NCT02228681 and NCT03660826 phase 2, NCT02684318 phase1/2,) a PTEN analysis is planned as part of secondary objectives. In a phase 2 trial (NCT00770185) the correlation between objective tumour response with PTEN expression is included in the primary objectives measurements [38].

## 5. Cervical Cancer

### 5.1. Incidence of PTEN Alteration

**Figure 4:**
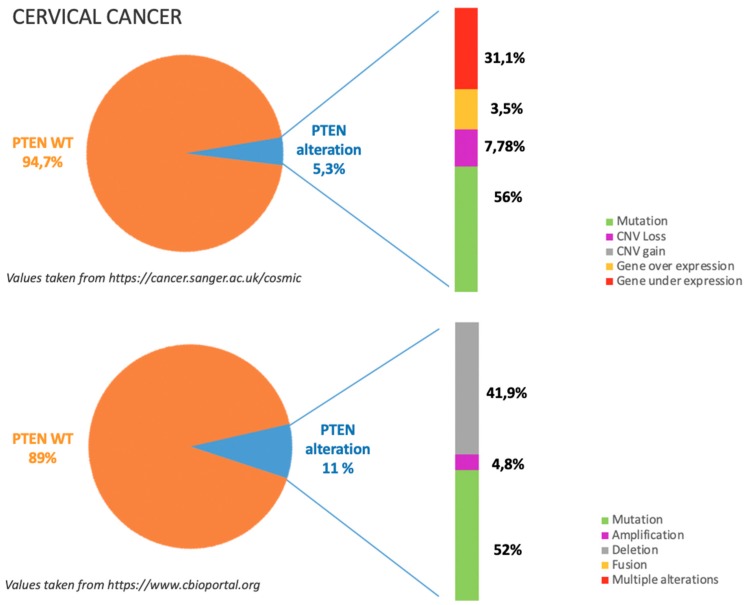
The incidence and type of PTEN alterations according to COSMIC and TGCA datasets in cervical cancer.

PTEN alterations in CC are around 8%, mostly due to missense and nonsense mutations as illustrated in Figure 4.

According to COSMIC dataset, see https://cancer.sanger.ac.uk/cosmic in Figure 4, gene mutations occur in almost half of the cases in which PTEN is altered; in particular among gene mutations missense substitutions are reported to be the most frequent (36%) followed by frameshift deletions (14%), and nonsense substitutions (28%). Moreover around 30% of PTEN alterations are related to gene under expression. This data is consistent with TGCA dataset, see https://www.cbioportal.org in Figure 4, which reports that among PTEN alterations half are due to gene mutation and around 40% are due to gene deletion [8,9,53,54].

Slightly different data come from less recent publications on Asiatic patients [53,54,55].

A group from Hong Kong analysed 10 high-grade cervical intraepithelial neoplasia and 62 squamous CC finding no PTEN mutation, a 13% rate of PTEN loss of heterozygosity and a 58% rate of PTEN promoter methylation [55].

Among 133 Indian CC cases, 23% showed loss of heterozygosity in at least one locus in the region examined of PTEN gene [56]. 

In 285 Chinese CC patients (179 squamous CC, 62 with adeno CC, 34 with adenosquamous CC, and 10 with other rare histopathological types) PTEN mutation rate analysed by Sanger sequencing was 2.8% [57].

These data discrepancies could be related to both technical (method used to detect PTEN status) and racial factors. Overall PTEN alterations in CC are not frequent but vary significantly among different histotypes.

The incidence of PTEN alterations is reported in Table 3 according to histological subtypes.

Adenocarcinoma and endocervical samples seems to show a higher rate of PTEN mutations compared to squamous histotype in both datasets.

### 5.2. PTEN Pathways in CC Tumorigenesis

Various reports demonstrated a possible implication of this protein in CC carcinogenesis. 

Data published in 2000 after screening 20 primary CC cases for PTEN loss of heterozygosity suggested that the disruption of PTEN by allelic loss or mutation may contribute to tumorigenesis in CCs [58].

It has been demonstrated that reduced PTEN expression progressively increase along the continuum from normal epithelium to squamous cell carcinoma (*p* < 0.01) [59]. 

Comparing premalignant lesions and invasive CC samples, the number of cases positive for PTEN immunostaining resulted significantly different (20/43, 46.5% vs. 24/105, 22.8% *p* = 0.005), suggesting that the loss of PTEN expression and activity could favour cervical carcinogenesis [60]. 

Similar results were published by a Chinese group comparing 42 cases of cervical adenocarcinoma, 20 cases of cervical glandular intraepithelial neoplasia and 28 cases of normal cervix tissue samples (23/42, 54.8% vs. 5/20, 25.0% vs. 28/28, 100%, respectively) [61].

Data published in 2015 demonstrated that EGF-dependent PI3K/AKT activation induces PTEN ubiquitination and destabilization leading to PTEN suppression and acceleration of CC tumourigenesis [62]. Moreover, in a Chinese study on 102 CC cases, PTEN promoter methylation was detected in 62% of cases in the tumour DNA and significantly correlated with the FIGO stage, carcinoma size, lymph node metastasis and tumour grade (*p* < 0.05), suggesting that it may play an important role in the occurrence and development of CC [63]. Similar data on PTEN correlation with FIGO stage, tumour size and prognosis had been already published on 50 primary CC cases [64]. 

Currently, ~99% of cases of CC are caused by high-risk Human Papilloma Virus (HPV) [65]. 

In a genomic characterization of 299 cases of CC, the authors found that together with TP53, ARID5B, ARID1A, and CTNNB1, PTEN was significantly differentially mutated between HPV+ and HPV− tumors (<15% vs. 33.33%, respectively) [66]. This may be related to the fact that HPV+ patients can slowly develop CC by accumulating somatic mutations in cancer driver genes while HPV− patients tend to accumulate higher levels of those mutations, more quickly.

This data keeps with previously published papers although the small sample size of HPV− CC makes it difficult to generalize results [58].

### 5.3. Clinical Implications

PTEN is an inclusion criterion in a phase 1 clinical trials including CC patients (NCT01226316, active not recruiting) and in a substudy of a phase 2 clinical trial (NCT02465060) [38]. 

Overall, when PTEN loss/mutation occurs in CC, it has been shown to aberrantly activate PI3K/AKT/mTOR pathway as expected. Therefore, further evaluation of a therapy regimen targeting downstream effectors such as PI3K or mTOR is warranted considering also it has already provided meaningful clinical benefits [67]. 

Recent data suggest that CC cell signalling through the PI3K/AKT pathway is distinct in obese (BMI ≥ 30) versus non-obese patients; in particular obese (BMI ≥ 30) CC patients with a PIK3CA and PTEN mutation fail to express high levels of phosphorylated AKT as expected [68]. This is associated with a trend toward favourable outcomes. 

## 6. Vulvar Cancer

### 6.1. Incidence of PTEN Alteration

**Figure 5:**
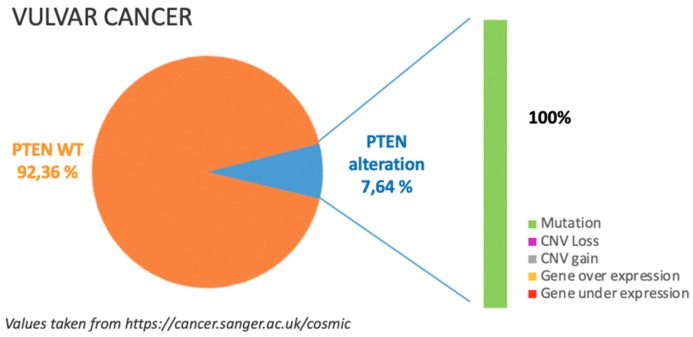
The incidence and type of PTEN alterations according to COSMIC dataset in vulvar cancer.

As previously mentioned, PTEN alterations in VC are around 7%, mostly due to missense substitution as illustrated in Figure 5.

Details are provided in Table 4. 

Only COSMIC dataset, see https://cancer.sanger.ac.uk/cosmic in Figure 5, is available for this type of cancer and all alterations are to be referred to gene mutations [8]. 

Recently, two groups published on molecular characteristics of VC according to HPV status, revealing a 9% (2/22) and 2% (1/52) rate, respectively, of PTEN mutations in HPV related tumours and 0% in HPV-tumours [69,70]. In both studies numbers were too low to get to definite conclusions but it is interesting to note that results are opposed to the trend observed in HPV+/HPV-CC cases.

Few data are available on other histologies rather than squamous one. 

The analysis of a series of 23 cases of adenocarcinoma VC, revealed a nearly 65% rate of PTEN loss measured by immunohistochemistry [71]. This rate was higher but not significantly different from the one obtained on squamous cancer samples. Almost half (43 and 46%) of the tumour samples utilized for testing were attained from metastatic sites. 

### 6.2. PTEN Pathways in VC Tumorigenesis

HPV oncogenic proteins have been shown to activate the PI3K pathway [72]. Data on 743 HPV-mediated cancers of which 83 were VC, showed that only five cases of PIK3CA-mutant VC were detected but co-occurrence of PTEN loss was interestingly high (80%) [73]. These samples were not regional metastases but primary tumours or distant metastasis. It remains unclear, however, whether this pathway significantly contributes to VC pathogenesis but it seems that PIK3CA mutations require a secondary defect in a co-regulator of PI3K activity, such as PTEN, to promote transformation. 

### 6.3. Clinical Implications

No studies are currently on going regarding PTEN mutations in VC [38].

## 7. Uterine Cancers

### 7.1. Incidence of PTEN Alteration

**Figure 6:**
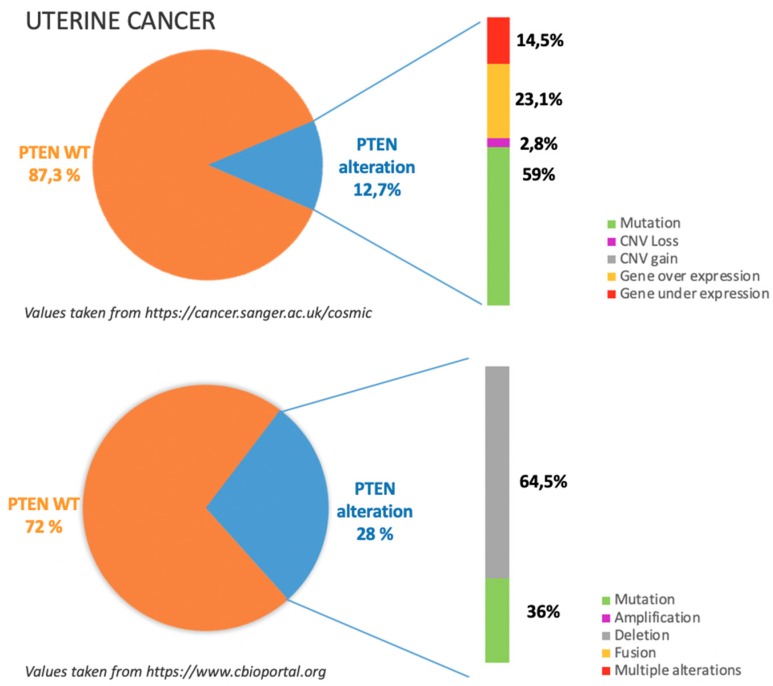
The incidence and type of PTEN alterations according to COSMIC and TGCA datasets in uterine cancer.

As previously mentioned, PTEN alterations in UC are overall reported to be around 20.5%, mostly due to copy number losses and missense and nonsense substitutions or deletions as illustrated in Figure 6.

According to COSMIC dataset, see https://cancer.sanger.ac.uk/cosmic in Figure 6, gene mutations occur in more than half of the cases in which PTEN is altered; in particular among gene mutations missense substitutions are reported to be the most frequent (66.67%) followed by frameshift deletions (25%) and nonsense substitutions (8.33%). Moreover about 30% of PTEN alterations are related to gene under or over expression. 

This data is not completely superimposable with TGCA dataset, see https://www.cbioportal.org in Figure 6, which reports that among PTEN alterations more than half are due to gene deletion and only 36% due to gene mutation [8,9].

Details are provided in Table 5. 

For leiomyosarcoma, there are no data available on COSMIC.

A more recent immunohistochemical analysis on 157 uterine leiomyosarcomas noted absence or low expression of PTEN in 28% of uterine leiomyosarcoma samples, coherently with other previously published data on soft tissue sarcomas and TGCA datasets [74,75,76,77,78].

Finally, an integrated genome analysis was performed on 84 uterine leiomyosarcoma samples (60 new samples and 24 from TGCA) by the same group, revealing a significantly higher rate of PTEN alterations (75% of samples), mainly due to gene deletions (71%) resulting in downregulation in 58% of cases [79]. These evidences suggest that previously published data may have underestimated PTEN alterations in uterine leiomyosarcoma. Given the rarity of this condition, more evidences are necessary to get to final conclusions.

### 7.2. PTEN Pathways in UC Tumorigenesis

The PI3K/AKT/mTOR pathway seems to play a key role in sarcomas pathogenesis, in line with evidences of dual PI3K/ mTOR inhibition efficacy in uterine leiomyosarcoma patient-derived xenografts [80]. However, PTEN role specifically remains unclear. 

A previous study suggested that the PTEN mutation might be a useful biomarker of cell proliferation in sarcomas [81]. 

### 7.3. Clinical Implications

PTEN is an inclusion criterion in two phase 2 clinical trials regarding leiomyosarcoma (NCT02987959, NCT03718091) [38].

## 8. Future Perspectives and Conclusions

Enormous effort is being been put into development of new strategies for gynaecological cancer patients. In the era of precision medicine, immunotherapy and PARP-inhibitors are gaining interest especially in these patients.

There is emerging evidence that target inhibition of PI3KCA/AKT/mTOR pathway can be combined with immunotherapy and/or PARP inhibitors to exploit potential synergy. 

As far as immunotherapy concerns, PTEN loss has been demonstrated to confer resistance to PD-L1 blockade in pre-clinical and clinical works.

On melanoma models the combined treatment with a PIK3b inhibitor and anti-PD-L1 resulted in enhanced tumour growth inhibition [82,83]. 

It has been suggested that PTEN can increase Treg stability and that the loss of PTEN can lead to spontaneous inflammatory disorder. A Chinese group recently published data on PTEN expression and CD4 + FOXP3 + T cells in 200 EC patients and 100 controls, showing that PTEN expression was negatively correlated with the count of CD4 + FOXP3 + T cells and that PTEN loss and the accumulation of CD4 + FOXP3 + T cells in lesions were inversely related to prognosis [84,85]. 

More recently, somatic PTEN mutations have been associated with resistance to immune checkpoint inhibitors by altering immunosuppressive environments in patients with uterine leiomyosarcoma, melanoma and glioblastomas [82,83,86]. 

Two clinical trials in solid malignancies, including gynaecological cancers, are currently combining immunotherapy and PI3K/Akt inhibitors (NCT03772561, phase 1 on going, NCT03842228 phase 1 on going) [38].

PTEN-deficient tumours may also be more sensitive to PARP inhibitors, due to PTEN’s role in genomic integrity by delaying G2/M-phase progression of damaged cells, thus allowing time for DNA double-strand breaks repair by homologous recombination [87]. Recent evidence suggests that, like BRCA mutant cells, PTEN mutant cells are also characterized by an increase in DNA double-strand breaks and defects in homologous recombination [88]. This seems an intriguing frontier both for the possible extension of Parp inhibitors indication to PTEN deficient patient and for the evaluation of the combination of Parp inhibitors and PI3K inhibitors. 

Two clinical trials in solid malignancies, including gynaecological cancers, have been designed to test the combination of PARP inhibitors and PI3K/Akt inhibitors (NCT02576444 phase 2, ongoing and NCT03842228 phase 1, to be started soon) while one to test the efficacy of PARP inhibitors in PTEN mutated patients (NCT02401347, phase 2, ongoing) [38].

Further studies to clinically characterize patients with PTEN alterations are warranted in order to offer potential approaches for development of personalized therapies.

It appears clear that only further integration of molecular, biological, and clinical information will allow scientists to keep the promise of precision medicine: customizing disease prevention and treatment strategies. 

## Figures and Tables

**Table 1:** Incidence of PTEN alterations in ovarian cancer.

Histology	Average %	N of Samples Tested	Details (Source: Number of Altered Samples/Total of Samples)
Malignant germ cell tumours (GCTs)	1.75	60	COSMIC dataset: 1/60 *
Sex cord-stromal tumour (SCSTs)	NA	36	COSMIC dataset **
Endometrioid	17.81	219	COSMIC dataset: 39/219
Clear cell	3.77	159	COSMIC dataset: 6/159
Mucinous	4.31	116	COSMIC dataset: 5/116
Low grade serous	NA	NA	COSMIC dataset
High grade serous carcinomas	5.4%	3667	COSMIC dataset: 92/2011 TGCA dataset: 108/1656
Undifferentiated carcinomas	NA	9	COSMIC dataset
Carcinosarcomas	6.98	43	COSMIC dataset: 3/43

NA: not available. * Histotypes included: 57 Teratoma, 1 Choriocarcinoma. ** Histotypes included: 34 Granulosa cell tumour, 1 Steroid cell tumour, 1 Sertoli–Leydig cell tumour.

**Table 2:** Incidence of PTEN alterations in endometrial cancer.

Histology	Average %	N of Samples Tested	Details (Source: Number of Altered Samples/Total of Samples)
Endometrioid	52.6	3319	COSMIC dataset: 1038/2092 TGCA dataset: 708/1227
Serous	8	467	COSMIC dataset: 10/144 TGCA dataset: 28/323
Clear cell	7.14	168	COSMIC dataset: 10/139 TGCA dataset: 2/29
Mixed	27	159	COSMIC dataset: 28/123 * TGCA dataset: 15/36
Dedifferentiated	36.36	22	COSMIC dataset: 8/22

* Histotypes included: 16 mixed endometrioid and clear cell carcinoma;26 Mixed cell carcinoma;81 Mixed serous and endometrioid.

**Table 3:** Incidence of PTEN alterations in cervical cancer according to histotypes.

Histology	Average %	N of Samples Tested	Details (Source: Number of Altered Samples/Total of Samples)
Squamous	6.7	1125	COSMIC dataset: 19/628 TGCA dataset: 57/497
Adenocarcinoma	6.25	112	COSMIC dataset: 5/81 TGCA dataset: 2/31
Endocervical	7.07	113	COSMIC dataset: 3/60 TGCA dataset: 5/53
Small cell	16.67	18	COSMIC dataset: 3/18
Endometrioid	22.22	9	COSMIC dataset: 2/9
Mixed adenosquamous	5.5	18	COSMIC dataset: 1/15 TGCA dataset:0/3

**Table 4:** Incidence of PTEN alterations in vulvar cancer according to histotypes.

Histology	Average %	N of Samples Tested	Details (Source: Number of Alterated Samples/Total of Samples)
Squamous	5.88	136	COSMIC dataset: 8/136

**Table 5:** Incidence of PTEN alterations in uterine cancers * according to histotypes.

Histology	Average %	N of Samples Tested	Details (Source: Number of Altered Samples/Total of Samples)
**Leiomyosarcoma**	70.37	27	TGCA dataset: 19/27
**Adenosarcoma**	18.18	11	COSMIC dataset: 2/11
**Carcinosarcoma/MMMT**	15.4	485	COSMIC dataset: 39/312 TGCA dataset: 36/173

* not including endometrial cancer.
